# Reverting estrogen-receptor-negative phenotype in HER-2-overexpressing advanced breast cancer patients exposed to trastuzumab plus chemotherapy

**DOI:** 10.1186/bcr1366

**Published:** 2005-12-07

**Authors:** Elisabetta Munzone, Giuseppe Curigliano, Andrea Rocca, Giuseppina Bonizzi, Giuseppe Renne, Aron Goldhirsch, Franco Nolè

**Affiliations:** 1 Department of Medicine, Division of Medical Oncology, European Institute of Oncology, Via Ripamonti 435, 20141 Milan, Italy; 2 Department of Experimental Oncology, European Institute of Oncology, Via Ripamonti 435, 20141 Milan, Italy; 3 Division of Pathology, European Institute of Oncology, Via Ripamonti 435, 20141 Milan, Italy

## Abstract

**Introduction:**

The amounts of estrogen receptor (ER) and progesterone receptor (PgR) in a primary tumor are predictive of the response to endocrine therapies of breast cancer. Several patients with ER-positive primary tumors relapse after adjuvant endocrine therapy with no ER expression in the recurrent tissue; much fewer with a recurrent disease after an ER-negative primary tumor may become endocrine responsive. These sequences of events indicate that a phenotype based on ER expression may not be a permanent feature of breast cancer.

**Methods:**

Ten patients with advanced breast cancer whose tumors overexpressed HER-2, but not ER or PgR, were treated with weekly trastuzumab at standard doses with or without chemotherapy.

**Results:**

Three out of 10 patients showed overexpression of ERs first appearing after 9, 12 and 37 weeks, respectively, from the initiation of trastuzumab. Two of these patients were subsequently treated with endocrine therapy alone: one of them received letrozole for 3 years without evidence of progression.

**Conclusion:**

Therapeutic targets enabling the appearance of an endocrine responsive disease may increase treatment options for patients with breast cancer. Furthermore, these clinical data suggest that an ER-negative phenotype is a multi-step process with a reversible repression modality, and that some ER-negative tumors may either revert to an ER-positive phenotype, allowing an endocrine treatment to be effective.

## Introduction

Biological features of endocrine responsiveness, namely the overexpression of estrogen receptor (ER) and/or progesterone receptor (PgR), are important predictive and prognostic markers in breast cancers [1]. About 50% of patients with ER-positive primary tumors that relapse after adjuvant tamoxifen therapy have recurrent tumors in which ER expression is lost [2]. Progression to an ER-negative phenotype typically involves the constitutive overexpression of growth-promoting genes that are normally regulated by estrogens, thereby leading to a loss of estrogen dependence, resistance to anti-estrogens, and a more aggressive phenotype overall.

The re-expression of ER in tumors in which ER has been lost or is not expressed could therefore indicate sensitivity to endocrine therapy. ER-negative tumors frequently overexpress growth factor receptors such as epidermal growth factor receptor (EGFR) or c-erbB2 [3,4], as do many ER-negative breast cancer cell lines, suggesting that upregulated growth factor signaling might provide an alternative growth stimulus. The overexpression of EGFR and c-erbB2 is also an important prognostic indicator of breast cancer, independently of their inverse correlation with ER expression [5,6]. This increased expression and/or activation of growth factor receptors correlates with increased mitogen-activated protein kinase (MAPK) activity, both in tumors and in cell lines [7-9]. Using cell lines that overexpress constitutively active forms of Raf-1, MAPK/ERK kinase (MEK)-1, or c-erbB-2 and ligand-activatable EGFR, some authors have shown that the resultant hyperactivation of MAPK (ERK1/2) activity through these signaling pathways leads to the downregulation of ERα. This downregulation is not a consequence of ligand-independent ERα activation and is reversible *in vitro*. Abrogation of ERK (extracellular signal-regulated kinase) activity with the use of either pharmacologic inhibitors or dominant-negative ERK constructs reverses the downregulation of ERα and restores its activity [10]. These data suggest that upregulated growth factor signaling via MAPK is directly linked to the loss of ER expression and generation of the ER-negative phenotype and that, at some stage in the progression pathway, the ER-negative phenotype may be not permanent.

As a consequence, therapeutic targets enabling the restoration of ER expression may provide beneficial treatment strategies for breast cancer patients.

Both gefitinib (Iressa^®^), which inhibits the kinase activity of EGFR, and trastuzumab (Herceptin™), a monoclonal antibody against c-erbB2, have been shown to reduce MAPK activity [11].

These data support the hypothesis that a patient with advanced breast cancer with ER-negative tumors and c-erbB2 overexpressed could revert from an ER-negative phenotype to ER-positive after treatment with trastuzumab.

## Methods

From August 1999 to November 2002, 10 patients with advanced breast cancer whose tumors overexpressed HER-2 (defined as a HercepTest score of 3+; Dako, Carpinteria, CA, USA), and with both ER and PgR negative were treated with weekly trastuzumab (Herceptin; Hoffman La-Roche, Basel, Switzerland) at a weekly dosage of 2 mg/kg intravenously (after an initial loading dose of 4 mg/kg) plus chemotherapy. Paclitaxel (Taxol^®^; Bristol-Myers Squibb Company, Princeton, NJ, USA) at a weekly dosage of 90 mg/m^2 ^intravenously, for three consecutive weeks every 4 weeks, was given to nine patients; the tenth patient received vinorelbine (Navelbine^®^; Pierre Fabre Pharma-30, Pierre Fabre Medicament-F-2849) at a weekly dosage of 20 mg/m^2 ^per week intravenously, for three consecutive weeks every 4 weeks.

Chemotherapy started concomitantly with trastuzumab in seven patients and, after evidence of progression, to trastuzumab alone in three patients. Tumor tissue for immunohistochemical analysis was obtained from all patients at baseline and after a median period of 9 weeks (range 3 to 37 weeks) of treatment with trastuzumab, with or without chemotherapy. All samples were processed and read in the same laboratory.

Basal evaluations were assessed on primary tumors in four patients and on metastatic sites in six (skin in four patients, pleura in one, liver in one). Tumor immunohistochemical reassessment was performed on the primary tumor in one patient, on the metastatic skin tissue in seven and on other metastatic sites in the remaining two (pleura in one, ovary in one) (Table 1).

Response to treatment was assessed in accordance with World Health Organization criteria.

The study protocol was reviewed and approved by the research ethics committee of the study center and relevant regulatory authorities. The study treatment was performed in accordance with Good Clinical Practice and the Helsinki declaration. Written informed consent was obtained from all patients.

## Results

We observed that 3 out of 10 patients had tumors that were ER positive (at least 10% of cells) after the histologic reassessment (see Table 1).

Patient 1 had a metastatic disease to bone, skin and liver and had a partial response to trastuzumab and paclitaxel after 1 month. Two months after treatment start she had a skin biopsy in the same site as baseline, with evidence of ER positivity (10%). The response to the combination chemotherapy and immunotherapy was maintained for 5 months. The patient was subsequently treated with multiple cycles of chemotherapy and died of progressive disease about 1 year later.

Patient 5 had metastatic disease to the liver and did not respond to trastuzumab. After 2 months from the start of trastuzumab treatment she underwent ovariectomy because of progressive disease. The ER and PgR assessment on ovarian metastases was positive (ER 50%; PgR 40%). Subsequently she received trastuzumab and paclitaxel without any response, and afterwards letrozole for 3 months with a progression to CNS (central nervous system). The patient died 6 months later.

Patient 6 underwent therapy with paclitaxel and trastuzumab for a cutaneous and controlateral relapse and had a partial response after 1 month of treatment. Seven months later she underwent mastectomy with evidence of positive ER. Subsequently the patient received letrozole for 3 years without progression of disease. Recently, she had a subcutaneous relapse that was biopsied and showed negative for ER and PgR, with HER-2 overexpressed; she is currently being treated with vinorelbine and trastuzumab.

In these three patients we observed the reversion of ER to a positive phenotype after 9, 12 and 37 weeks, respectively, from the start of treatment with trastuzumab. In two patients (patients 1 and 6) trastuzumab was given concomitantly with paclitaxel; the third (patient 5) had received trastuzumab alone when ER re-expression was assessed.

## Discussion

This is the first report of an ER-negative phenotype changing to ER positive after a trastuzumab-containing therapy in patients with advanced breast cancer. Interestingly, previous reports evaluating the use of trastuzumab in the preoperative setting in patients with locally advanced breast cancer do not address this particular issue [12,13].

The patient sample was limited, but this could be an important anecdoctal and hypothesis-generating report that should be confirmed in larger trials.

Another potential limit was that the reassessment was performed at a variable time point after the start of therapy, and the timing was not predefined. Our three patients had a reversion of ER to a positive phenotype after 9, 12 and 37 weeks from the start of treatment with trastuzumab. For this reason we do not have data on which to base a hypothesis about the optimal timing for performing the reassessment.

A recent report by Arpino *et al*. proposed that a lack of PgR expression could be a reflection of active signaling in the HER family and, as a consequence, the response to trastuzumab might be different in the PgR-positive and PgR-negative subsets [14]. Interestingly, two of our patients after ER reversion to a positive phenotype remained PgR negative; both had a partial response to trastuzumab, whereas the only patient who also had a PgR reversion was resistant to trastuzumab.

Other studies that address ER loss reversibility in breast cancer cell lines involve treatments with demethylating agents such as 5-azacytidine [15,16]. About 25% of ER-negative breast tumors were found to contain methylated ER [17]. However, several ER-positive tumors also showed similar degrees of methylation [18]. Methylation, by modifying the DNA structure assisting in the recruitment of histone deacetylases, ensures that the gene is in an inactive conformation. If methylation occurs as a step subsequent to another mechanism of ER repression, one would predict that some ER-negative breast tumors at an earlier point in a progression pathway do not express ER because one of these other mechanisms is still operative. In such tumors, it might then be possible to reverse this phenotype and restore antiestrogen sensitivity, perhaps by using a demethylating agent.

An interesting recently reported transcription factor that might be linked to hormone-independent breast cancer, as well as elevated growth factor signaling, is NF-κB. Its activity is elevated in hormone-independent breast cancer, and it is implicated in enhanced cell survival and chemoresistance in cancer [19]. Holloway *et al*. have shown recently that an elevation in NF-κB activity is attributable to enhanced growth factor signaling through ERK1/2; inhibiting this NF-κB activity, either pharmacologically or through the expression of a constitutively active IκB, partly restores ER activity and expression in these cells. These findings suggest a role for cytoplasmic substrates of MAPK in ER downregulation in breast cancer and further support a role for MAPK-induced NF-κB activity in this downregulation [20].

## Conclusion

We conclude that our clinical data – although limited in the patient sample – support previous assessments *in vitro*, suggesting that an ER-negative phenotype may be a multi-step process with a reversible repression modality, and that some ER-negative tumors might revert to the ER-positive phenotype allowing an endocrine-based treatment.

Although further studies are essential to show that inhibitors of mitogenic signaling can restore ER expression and/or function in breast tumors, coupling molecules such as signal transduction inhibitors, monoclonal antibodies, demethylating agents or NF-κB inhibitors with antiestrogens could result in therapies that are better tolerated and active for the treatment of ER-negative breast cancer. In addition, once the MAPK substrate or substrates responsible for ER downregulation are identified, they could provide an additional drug target.

## Abbreviations

EGFR = epidermal growth factor receptor; ER = estrogen receptor; ERK = extracellular signal-regulated kinase; MAPK = mitogen-activated protein kinase; NF-κB = nuclear factor-κB; PgR = progesterone receptor.

## Competing Interests

The authors declare they have no competing intrests.

## Authors' contributions

EM made substantial contributions to the conception and design of the study, the acquisition of data and the analysis and interpretation of data, and drafted the manuscript. GC made substantial contributions to the conception and design of the study, the acquisition of data and the analysis and interpretation of data, and contributed to drafting the manuscript. AR made substantial contributions to the conception and design of the study and contributed to drafting the manuscript. GB made substantial contributions to the conception and design of the study and to the analysis and interpretation of data. GR processed and read all histological samples and performed the immunohistochemistry. AG made substantial contributions to the conception and design of the study and gave the final approval of the version to be published. FN made substantial contributions to the conception and design of the study, the acquisition of data and the analysis and interpretation of data. All authors read and approved the final manuscript.

## Figures and Tables

**Table 1:** Patients' characteristics

Patient	Baseline evaluation					After treatment							
	ER, %	PgR, %	HER2, % (score)	First assessment	Time A	Chemotherapy	Time B	Second assessment	ER, %	PgR, %	HER2, % (score)	HT	Clinical response
1	0	0	80 (3+)	Skin	20	Paclitaxel	9	Skin	10	0	70 (3+)	No	PR
2	0	0	70 (3+)	Pleura	87	Paclitaxel	11	Pleura	0	0	75 (3+)	No	SD
3	0	0	90 (3+)	Skin	8	None^a^	4	Skin	0	0	90 (3+)	No	PR
4	0	0	40 (3+)	Skin	31	Paclitaxel	9	Skin	0	0	90 (3+)	No	PD
5	0	0	90 (3+)	Liver	5	None^a^	12	Ovary	50	40	80 (3+)	Yes	PD
6	0	0	95 (3+)	Breast	2	Paclitaxel	37	Breast	20	0	90 (3+)	Yes	PR
7	0	0	80 (3+)	Skin	64	None^a^	4	Skin	0	0	95 (3+)	No	PR
8	0	0	90 (3+)	Lung	2	Paclitaxel	4	Skin	0	0	90 (3+)	No	SD
9	0	0	80 (3+)	Skin	51	Paclitaxel	22	Skin	0	0	40 (2+)	No	PR
10	0	0	90 (3+)	Skin	17	Vinorelbine	3	Skin	0	0	90 (3+)	No	PR

The table shows the evaluation of patients' characteristics at baseline with the site of disease at which the biological characteristics were evaluated, and after treatment with trastuzumab with the site of disease at which the biological characteristics were reassessed. ER, estrogen receptor; first assessment, site of disease where ER, PgR and HER2 were evaluated at baseline; HT, hormonal treatment; PgR, progesterone receptor; second assessment, site of disease where ER, PgR and HER2 were reassessed; time A, time elapsed between first assessment and treatment start (weeks); time B, time elapsed between trastuzumab start and second assessment (weeks). PD, progressive disease. SD, stable disease. PR, partial response.

^a^Paclitaxel was introduced after progression to trastuzumab alone.
